# Analysis of distribution equilibrium and influencing factors for older adult meal service facilities in mainland China

**DOI:** 10.3389/fpubh.2025.1578827

**Published:** 2025-06-25

**Authors:** Feng Wang, Hengzhu Zhou, Chengcheng Lv, Xiaokang Song

**Affiliations:** School of Management, Xuzhou Medical University, Xuzhou, China

**Keywords:** older adult meal service, distribution equilibrium, Spatial Mismatch Index, Gini coefficient, influencing factors

## Abstract

**Objective:**

Analyze the distribution equilibrium of older adult meal service facilities in mainland China and explore the factors influencing their distribution.

**Methods:**

Use Python to obtain data on older adult meal service facilities, and analyze the equity of older adult meal services using descriptive statistics, the Lorenz curve, the Gini coefficient, and the Spatial Mismatch Index (SMI). A multiple linear regression model was applied to explore the relationships between older adult meal services and factors such as population, economy, infrastructure, geography, and policies.

**Results:**

The vast majority (85.26%) of older adult meal service facilities in mainland China are located at the community or village level, with the number of rural facilities significantly higher than that in urban areas. Most of these facilities are self-operated, and those with in-house kitchens dominate. The distribution of older adult meal services across mainland China was uneven, with a Gini coefficient of 0.418, indicating a substantial disparity in the allocation of service resources. The Spatial Mismatch Index (SMI) varied significantly among provinces, reflecting a considerable mismatch between the current supply of older adult meal service facilities and the actual demand of the older adult population. Analysis of influencing factors reveals that the number and coverage level of older adult meal service facilities were significantly negatively correlated with the size of the older adult population, the older adult dependency ratio, and GDP.

**Conclusion:**

The equity of older adult meal services still needs improvement. The planning and implementation of meal services should place greater emphasis on population demand to ensure that older adult individuals across the country can fairly access the older adult meal services they deserve. Future policy formulation should comprehensively consider factors such as demographic structure, economic development levels, and infrastructure conditions to optimize resource allocation, reduce regional disparities, and enhance service coverage and quality.

## 1 Introduction

According to the 2023 National Report on the Development of Aging Affairs (1), by the end of that year, the older adult population aged 65 and above in China had reached 216.76 million, accounting for 15.4% of the total population. The average life expectancy in China has shown a year-on-year increasing trend, and it is predicted that by 2035, the life expectancy at birth in mainland China will rise to 81.3 years (2). With the accelerated development of an aging society and the growth in average life expectancy, the challenges faced by the older adult care service industry are becoming increasingly severe. These challenges are multifaceted and include labor shortages, weak quality supervision, insufficient funding, significant urban–rural disparities, imbalanced service models, and inadequate insurance coverage (3–5).

Chinese older adult exhibit a strong preference for aging in place, with over 90% of older adults inclined to spend their retirement years at home (6). Community Home-based Elderly Care Services (CHECS), as a crucial support model for aging, have been proven to effectively enhance life satisfaction among the older adult and significantly improve their self-reported health status (7, 8). Notably, on October 20, 2023, China officially released its first national standard for home-based older adult care services (9). This standard outlines seven key service components, including life care, basic care, health management, visitation and care, emotional support, proxy services, and home environment adaptation for aging, aiming to provide a safe and comfortable living environment for older adults.

Maslow’s hierarchy of needs theory posits that food, as a basic physiological need at the first level, is the most fundamental and prioritized human requirement (10). Research by Wang et al. demonstrates that the dietary experiences of older adults have a significantly positive impact on their health, as “eating” can bring them joy (11). To meet this need among older adults and promote longevity while reducing the incidence of chronic diseases, healthy eating plays an indispensable role (12). Home-delivered meal services or communal dining activities are crucial for ensuring that older adults receive adequate nutrition (13, 14). For instance, a study by Seo et al. (15) found that through communal meal programs, older adult individuals reported high satisfaction even with reduced salt intake. Additionally, older adult meal services not only significantly reduce the risk of falls (16) but also alleviate loneliness and enhance social networks and well-being among older adults (17, 18). Therefore, older adult meal service facilities play a vital role in improving nutritional intake, reducing chronic diseases, and enhancing mental health.

The Chinese government is currently promoting the nationwide implementation of nutritious, affordable, and easily accessible welfare meal services for older adults to support aging in place and improve the quality of life for older adults (19). However, existing research primarily focuses on the impact of meal assistance services on the health status of older adults (11–18), while studies on the distribution equilibrium of these services and their influencing factors remain relatively insufficient. Therefore, this study aimed to explore the distribution equilibrium of older adult meal service facilities and its influencing factors, with the goal of providing scientific evidence for policymakers. By doing so, it seeks to promote the rational allocation of older adult care service resources and ensure that all older adults can equally access high-quality older adult care services.

## 2 Literature review

Older adult meal service is a service designed to provide dietary support for older adults, aiming to meet their nutritional needs, improve their quality of life, and alleviate the caregiving burden on families and society (20). Similar meal service models exist in other countries. For example, in the United States, programs such as Meals on Wheels (MOW) and the Congregate Meal Program offer home-delivered meals or communal dining services for older adults (21, 22). South Korea also has congregate meal programs (15). Similarly, countries like South Africa provide meal support for older adults through community-based meal programs (23). These services shared functional and goal-oriented similarities with China’s older adult meal service initiatives, as they all strive to address the dietary challenges faced by older adults and enhance their overall quality of life.

Although the importance of older adult meal services has been widely recognized—such as improving health and nutrition (13, 14), reducing the incidence of chronic diseases (12), lowering the risk of falls (16), and enhancing the well-being of older adults (17, 18) —significant disparities still exist in the distribution of older adult care resources and service efficiency across different regions. Zhang’s research, utilizing the Gini coefficient and Theil index, highlighted the uneven development of older adult care services in China, with an imbalanced allocation of older adult care resources (24). Specifically, the majority of older adult care resources are concentrated in the eastern regions, while the western and some central regions have relatively fewer resources available for older adult care. Yu further revealed substantial disparities in the distribution of older adult care resources among different provinces (regions and municipalities) using cluster analysis (25). From an economic perspective, the relatively abundant older adult care resources in economically developed provinces are primarily due to the high demand for such services by residents in these regions. These studies demonstrate that the overall equity of older adult care services is crucial for social development and addressing the challenges of an aging population.

Existing research has predominantly focused on the overall allocation of older adult care service resources (24, 25), while the equity of diet-related older adult meal services has received insufficient attention. This oversight might have resulted in older adults in certain regions being unable to access essential older adult care services to meet their basic physiological needs, thereby negatively impacting their health and quality of life. Therefore, based on data from the National Elderly Care Service Information Platform, this study conducted an equity analysis of older adult meal service facilities across mainland China and preliminarily explored the factors influencing their quantity and coverage.

## 3 Data sources and research methods

### 3.1 Research framework

**Figure 1:**
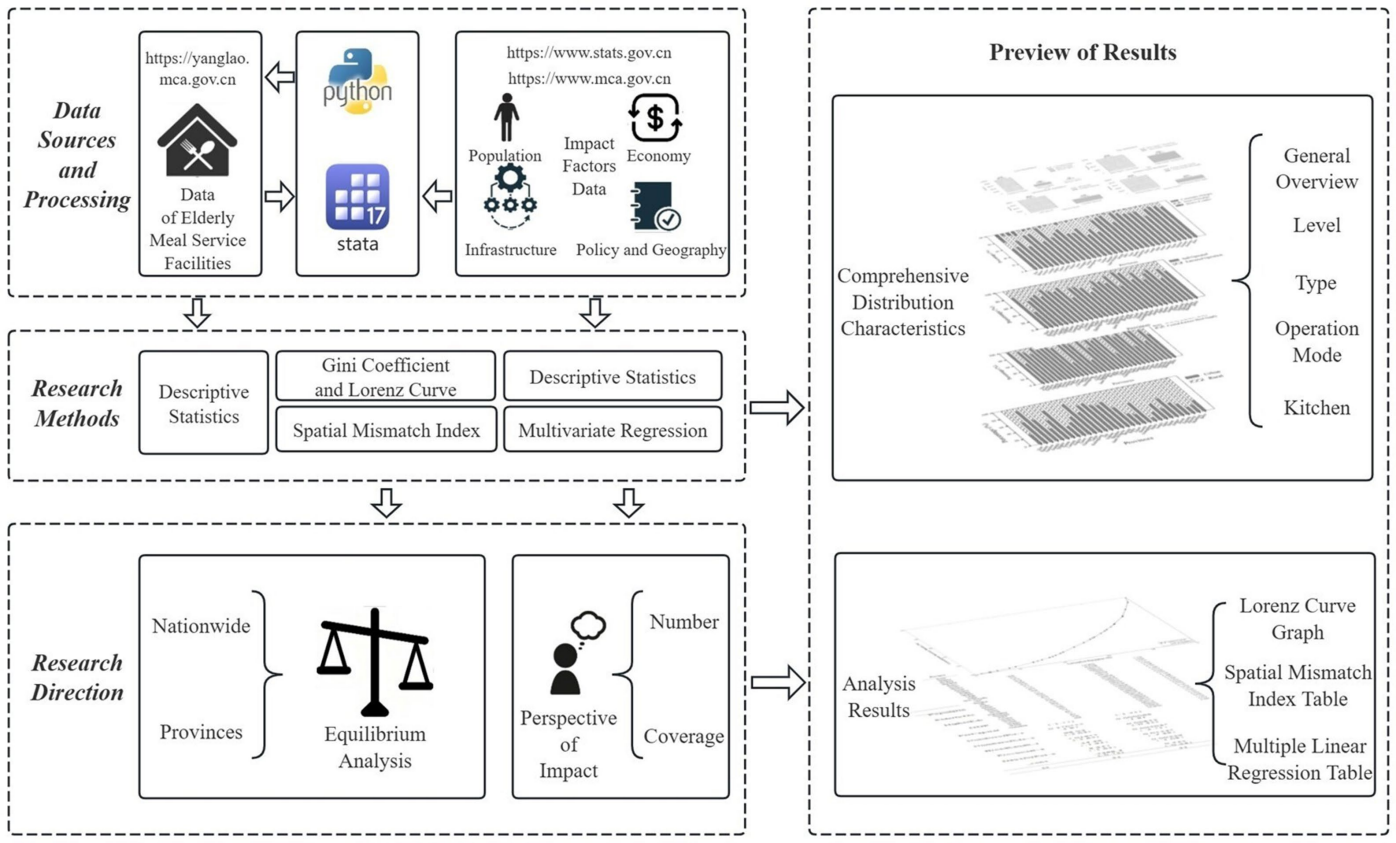
Research framework (source: original work).

This study primarily focused on the equity of older adult meal service facilities, aiming to identify and analyze the key factors influencing their distribution. As shown in Figure 1, the first step involves data sources and processing. This study obtained detailed data on older adult meal service facilities through Python programming from the National Elderly Care Service Information Platform and collected relevant socio-economic influencing factors from the National Bureau of Statistics. After acquiring these foundational datasets, this study conducted descriptive statistical analyses on the number and distribution of older adult meal service facilities to gain a preliminary understanding of their basic characteristics.

This study employed the classical supply–demand theory in economics as its theoretical framework, building upon Smith’s core concept of the market’s “invisible hand” that regulated supply–demand equilibrium, and Marshall’s subsequent development of supply–demand curves and equilibrium price models that established the theoretical foundation for resource allocation (26, 27). Focusing on the interaction between facility supply and older adult population demand, the research quantified supply–demand imbalance using the Lorenz curve and Gini coefficient, while introducing the Spatial Mismatch Index (SMI) to identify provincial-level supply-demand mismatches. Furthermore, it employed multiple linear regression analysis to reveal demand relationships.

Building on this, this study conducted a multiple linear regression analysis to examine the relationship between the collected influencing factors and the quantity and coverage level of older adult meal service facilities. Through this analysis, this study revealed the impact of various independent factors on the quantity and coverage level of older adult meal service facilities.

### 3.2 Data sources

To build a comprehensive national database of older adult meal service facilities, this study utilized the Python programming language to collect data from the National Elderly Care Service Information Platform^1^ as of December 19, 2024. The data collection process resulted in a total of 106,930 records of older adult meal service facilities. To ensure data quality and completeness, this study systematically preprocessed the raw data.

During the initial data cleaning process, this study identified 568 samples with missing address information. To address this issue, this study first utilized the MID function in Excel to parse the existing address fields and extract province information. For samples with incomplete address information, this study further supplemented the data by leveraging their geographic coordinates and applying the reverse geocoding service provided by the Amap API, successfully filling in the province information for 481 samples. For the remaining 87 samples, when geographic coordinates were unavailable, this study adopted a manual intervention approach by querying specific address information through the Baidu search engine, thereby determining the province for 68 of these samples. Considering the importance of data completeness and accuracy, this study ultimately decided to remove 19 records that could not be matched with accurate information from the dataset.

Therefore, after a series of data cleaning and completion efforts, this study ultimately retained 106,911 valid records of older adult meal service facilities. These data cover four key attributes of the facilities: level, type, operational model, and kitchen facilities, providing a solid foundation for subsequent research and analysis.

This study also obtained data on the older adult population, provincial area, and other relevant influencing factors from the official statistical database published by the National Bureau of Statistics of China^2^.

### 3.3 Research methods

#### 3.3.1 Descriptive statistics

Descriptive statistics were conducted on the four attributes of the national older adult meal service facilities (level, type, operational model, and kitchen facilities). The distribution of older adult meal service facilities across provinces was visually represented using bar charts, stacked bar charts, and percentage stacked bar charts.

#### 3.3.2 Lorenz curve and Gini coefficient

The Lorenz curve is a graphical tool used to illustrate the distribution of income or wealth among members of a society (28, 29), while the Gini coefficient is a numerical value derived from the Lorenz curve (30), quantifying the degree of inequality in this distribution, which can also be interpreted as an indicator of distribution equilibrium. In this study, the Lorenz curve and Gini coefficient were employed as analytical tools. By sorting the ratio of older adult population to older adult meal service facilities across regions, a Lorenz curve was constructed, visually depicting the cumulative percentage of the older adult population against the cumulative percentage of older adult meal service facilities.

The specific formulas are as follows:


(1) $$R_{i}=T_{i}/P_{i}$$

(2) $$T(x)=\frac{\sum_{i=1}^{x}T_{i}}{\sum_{i=1}^{n}T_{i}}$$

(3) $$\begin{array}{c} P(x)=\frac{\sum_{i=1}^{x}P_{i}}{\sum_{i=1}^{n}P_{i}} \end{array}$$

(4) $$\begin{array}{c} G=1-2\times \int_{0}^{1}\;T(x)\mathrm{dx} \end{array}$$

$R_{i}$ represents the ratio of the number of older adult meal service facilities (*T_i_*) to the population aged 65 and above (*P_i_*) in the i -th province. Based on the sorted order of *Ri*, the cumulative proportion of older adult meal service facilities T(x) in Equation (2) and the cumulative population proportion P(x) in Equation (3) are calculated. The Lorenz curve is then plotted using P(x) and T(x) , and the Gini coefficient G in Equation (4) is computed using numerical integration methods to assess the distribution equilibrium of older adult meal service facilities.

#### 3.3.3 Spatial Mismatch Index

The Spatial Mismatch Index (SMI) is an indicator used to measure the degree of mismatch between resource allocation and demand within a geographic region (31). It is commonly employed to assess whether the spatial distribution of a specific resource [such as job opportunities (32), medical services (33), etc.] aligns with the distribution of the population in need of these resources. In this study, the SMI is used to analyze whether the distribution of older adult meal service facilities matches the demand of the population aged 65 and above.

The formula for SMI is as follows:


(5) $$\begin{array}{c} \mathrm{SMI}=(\frac{T_{i}}{\sum_{i=1}^{n}T_{i}}-\frac{P_{i}}{\sum_{i=1}^{n}P_{i}})\times 100 \end{array}$$

(6) $$\begin{array}{c} \sum \mathrm{SMI}=\sum_{i=1}^{n}\;|{\mathrm{SMI}}_{i}| \end{array}$$

Here, in Equation (5) the ratio $\frac{T_{i}}{\sum_{i=1}^{n}T_{i}}$ indicates the proportion of older adult meal service facilities in the i -th province relative to the total number of facilities nationwide, and $\frac{P_{i}}{\sum_{i=1}^{n}P_{i}}$ represents the proportion of the population aged 65 and above in the i -th province relative to the total older adult population nationwide.

In Equation (6), ∑SMI reflects the overall level of spatial mismatch between older adult meal service facilities and the population aged 65 and above. Positive SMI values: Indicate that the province has more older adult meal service facilities relative to its population demand, suggesting potential over-supply or over-concentration of facilities. Negative SMI values: Indicate that the province has fewer older adult meal service facilities relative to its population demand, implying potential under-supply or uneven distribution of facilities. SMI values close to zero: Suggest that the province has a well-matched proportion of older adult meal service facilities to its population demand, indicating good spatial alignment between the two.

#### 3.3.4 Multivariate regression

This study aims to analyze the influencing factors of both the quantity and coverage level ( CL ) of older adult meal service facilities using multiple linear regression models (34). This study defines the formula for calculating the coverage level of older adult meal service facilities and apply multiple linear regression models to identify and quantify these influencing factors.

The formula for the coverage level of older adult meal service facilities is as follows Equation (7):


(7) $$\begin{array}{c} C=(\frac{T_{i}}{P_{i}}\frac{\sum_{i=1}^{n}P_{i}}{\sum_{i=1}^{n}T_{i}})\times 100 \end{array}$$

The formula for the multiple linear regression model is as follows:


(8) $$\begin{array}{c} Q=\beta_{0}+\beta_{1}X_{1}+\beta_{2}X_{2}+\ldots +\beta_{n}X_{n}+\in \end{array}$$

(9) $$\begin{array}{c} \mathrm{CL}=\beta_{0}+\beta_{1}X_{1}+\beta_{2}X_{2}+\ldots +\beta_{n}X_{n}+ \end{array}\in$$

Here, Q in Equation (8) represents the quantity of older adult meal service facilities, CL in Equation (9) represents the coverage level of older adult meal service facilities, $X_{1},X_{2},\ldots ,X_{n}$ denote the independent variables, $\beta_{0}$ is the intercept term, $\beta_{1},\beta_{2},\ldots ,\beta_{n}$ are the regression coefficients corresponding to each independent variable, and ϵ is the error term. The specific independent variable descriptions are shown in Table 1.

**Table 1:** Description of independent variables.

Variable	Name	Unit	Description
X1	Population Aged 65 and Above	10,000 persons	Number of people aged 65 and above in each province
X2	Older adult dependency ratio	%	Ratio of older adult population to working-age population, indicating the number of older adult persons per 100 working-age population
X3	Higher Education Enrollment	Persons	Number of people whose highest education level is junior college
X4	GDP	100 million yuan	Gross Domestic Product of each province
X5	GDP *Per Capita*	10,000 yuan	GDP divided by the average population
X6	Number of Public Buses and Electric Vehicles in Operation	Units	Total urban public transport vehicles (buses and electric vehicles) for passenger transport operations
X7	Number of Medical Institutions	1,000 units	Healthcare institutions including hospitals, primary care institutions, professional public health institutions, and others
X8	Broadband Subscriber Count	10,000 households	Registered users accessing the internet via xDSL, FTTX+LAN, WLAN, etc., including xDSL users, LAN terminal users, and wireless users
X9	Provincial Area	10,000 km^2^	Land area of each province
X10	Days for Policy Response	Days	Time interval between State Council directives and provincial policy releases

## 4 Comprehensive distribution characteristics of older adult meal service facilities

### 4.1 General overview

**Figure 2:**
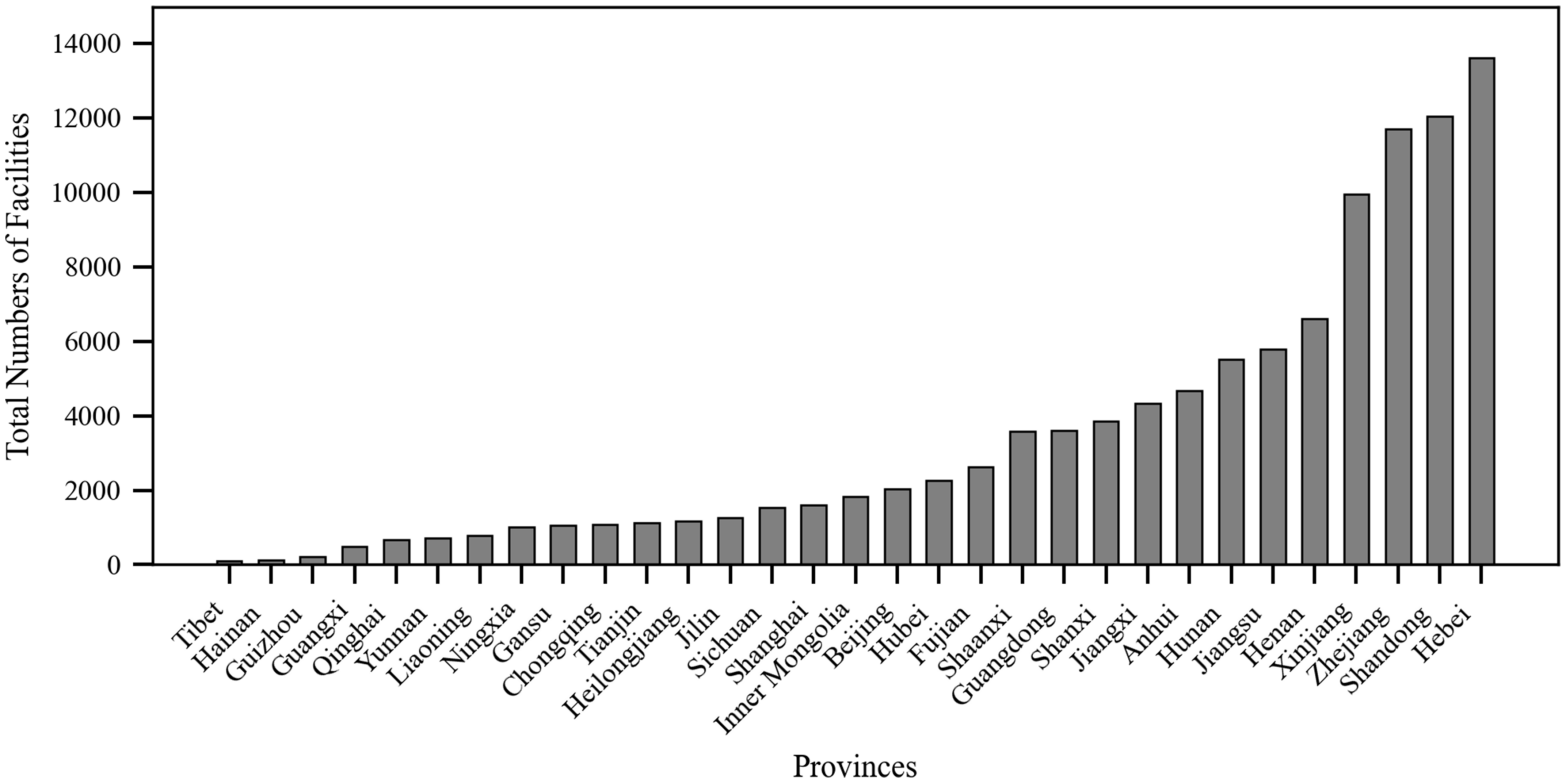
Total number of meal service facilities by province (source: original work).

According to data from the National Elderly Care Service Information Platform (See text footnote 1) as of December 19, 2024, there are a total of 106,911 older adult meal service facilities nationwide in China. As shown in Figure 2, there are significant disparities in the number of older adult meal service facilities among different provinces. Hebei Province has the highest number of facilities, totaling 13,610, while the Tibet Autonomous Region has only 112, reflecting a substantial gap. This may be related to the geographical location and population distribution of the provinces. Hebei, adjacent to Beijing, not only provides older adult meal assistance services for its local residents but also caters to seniors from Beijing. In contrast, Tibet’s vast territory and sparse population make it difficult to establish concentrated older adult dining services with agglomeration effects.

The total number of older adult meal service facilities in the eastern coastal regions was generally higher than that in the western inland provinces, which may be attributed to the higher economic development levels, population density, and greater demand for older adult care services in the eastern regions (35, 36). Beijing and Shanghai, as municipalities directly under the central government, had 2,038 and 1,604 older adult meal service facilities, respectively, despite their limited geographical areas, demonstrating these cities’ strong focus on older adult care services. Additionally, Henan and Shandong, as populous provinces in China, also have relatively high numbers of older adult meal service facilities, with 6,602 and 12,046 facilities, respectively.

**Figure 3:**
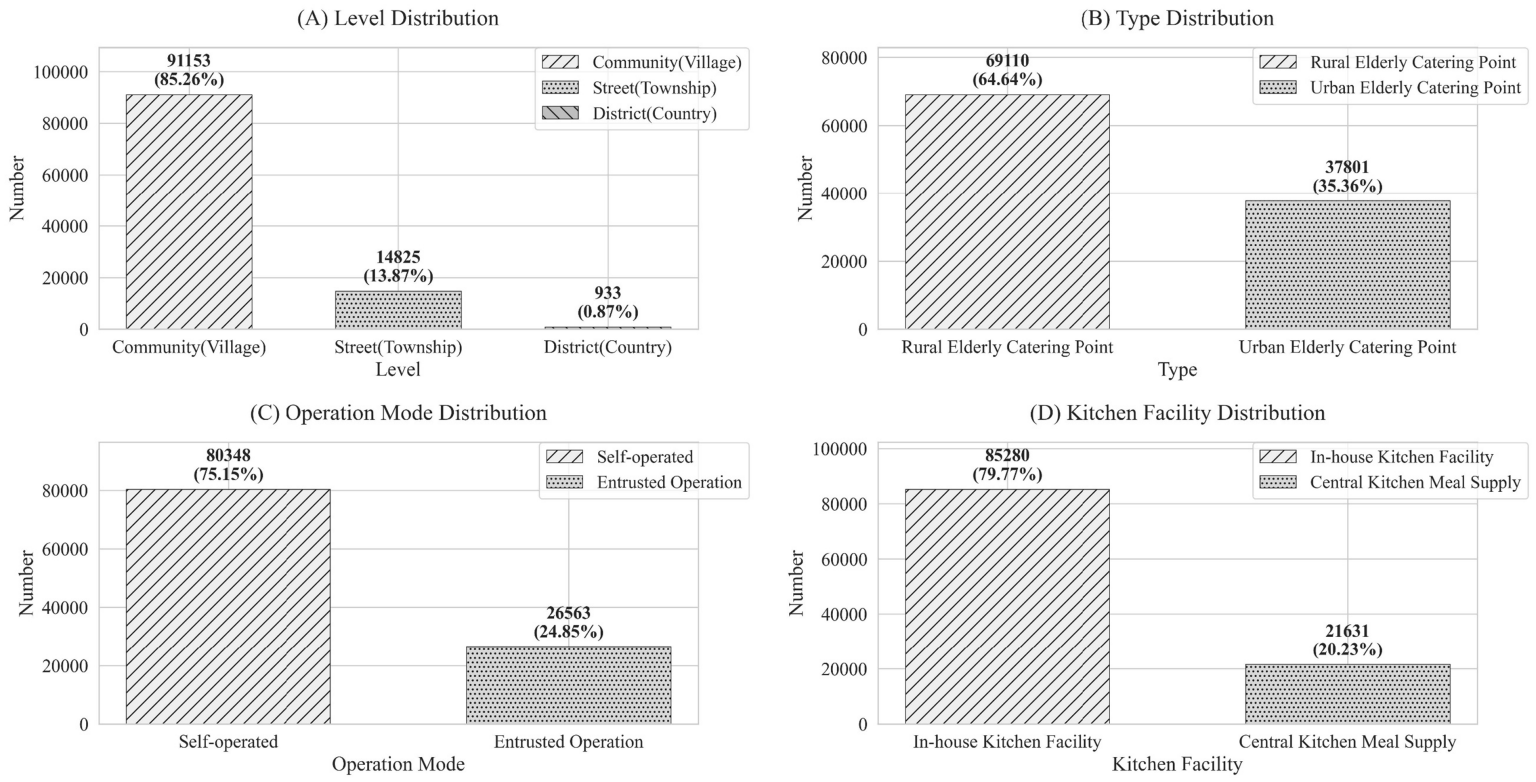
Comprehensive distribution characteristics of national older adult meal service facilities (source: original work).

Based on the four main characteristics of older adult meal service facilities—level, type, operational model, and kitchen facilities—this study could further analyze their distribution, as shown in Figure 3.

Level: The vast majority (85.26%) of facilities were located at the community or village level, totaling 91,153 facilities. Facilities at the township or street level accounted for 13.87% of the total, with 14,825 facilities, while district or county-level facilities were relatively few, making up only 0.87% with 933 facilities.

Type: The number of rural older adult meal service facilities was significantly higher than that in urban areas, with 69,110 facilities (64.64%) in rural areas and 37,801 facilities (35.36%) in urban areas. This reflected the greater demand for older adult care services in rural regions.

Operational Model: Most facilities operated independently, meaning the facility and its operating entity are integrated. There were 80,348 such facilities, accounting for 75.15% of the total. In contrast, 26,563 facilities (24.85%) were outsourced to external organizations for operation.

Kitchen Facilities: Facilities with on-site kitchens dominated, totaling 85,280 facilities (79.77%). Meanwhile, 21,631 facilities (20.23%) relied on central kitchens for meal preparation.

The above data provided a nationwide overview. To gain a deeper understanding of the specific situations in different regions, this study conducted a detailed analysis of older adult meal service facilities based on provincial administrative divisions. Through this level of analysis, this study aimed to reveal the disparities in the distribution of older adult meal service facilities among different provinces.

### 4.2 Meal service facility levels

**Figure 4:**
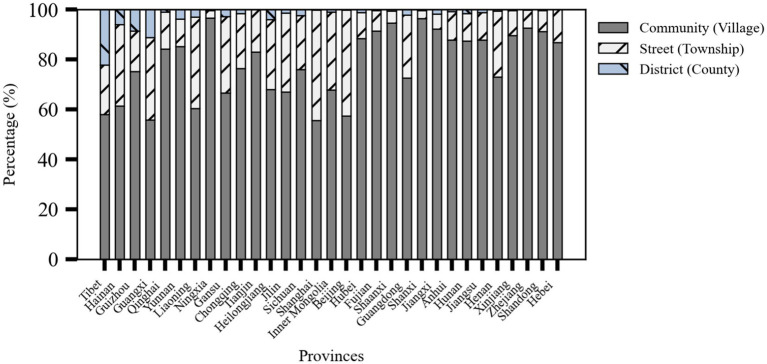
Stacked percentage bar chart of meal service facility levels by province (source: original work).

The older adult meal service facilities are categorized into three levels: community (village) level, township (street) level, and district (county) level, as shown in Figure 4. In terms of the hierarchical structure, the majority of provinces have the highest number of community (village)-level facilities, followed by township (street)-level facilities, and finally district (county)-level facilities. Provinces with a high proportion of community (village)-level facilities include Ningxia (96.53%), Shanxi (96.39%), and Shaanxi (94.60%), reflecting the extensive grassroots service networks. In contrast, more urbanized eastern regions like Shanghai (44.26%), Beijing (42.35%), and Liaoning (36.76%) have higher proportions of township-level facilities catering to concentrated residential areas. Meanwhile, with complex geography and less developed economies such as Tibet (22.32%), Guangxi (11.16%), and Guizhou (8.74%) rely more on county-level service facilities.

### 4.3 Meal service facility types

The older adult meal service facilities are categorized into rural and urban types. Provinces with a high proportion of rural older adult meal service facilities included Shanxi (90.22%), Zhejiang (82.15%), and Shandong (80.01%). Meanwhile, provinces with a high proportion of urban facilities included Guizhou (91.26%), Tianjin (84.75%), and Liaoning (79.82%). These patterns may be closely related to the geographical environment, population distribution structure, and regional development policies of each area.

**Figure 5:**
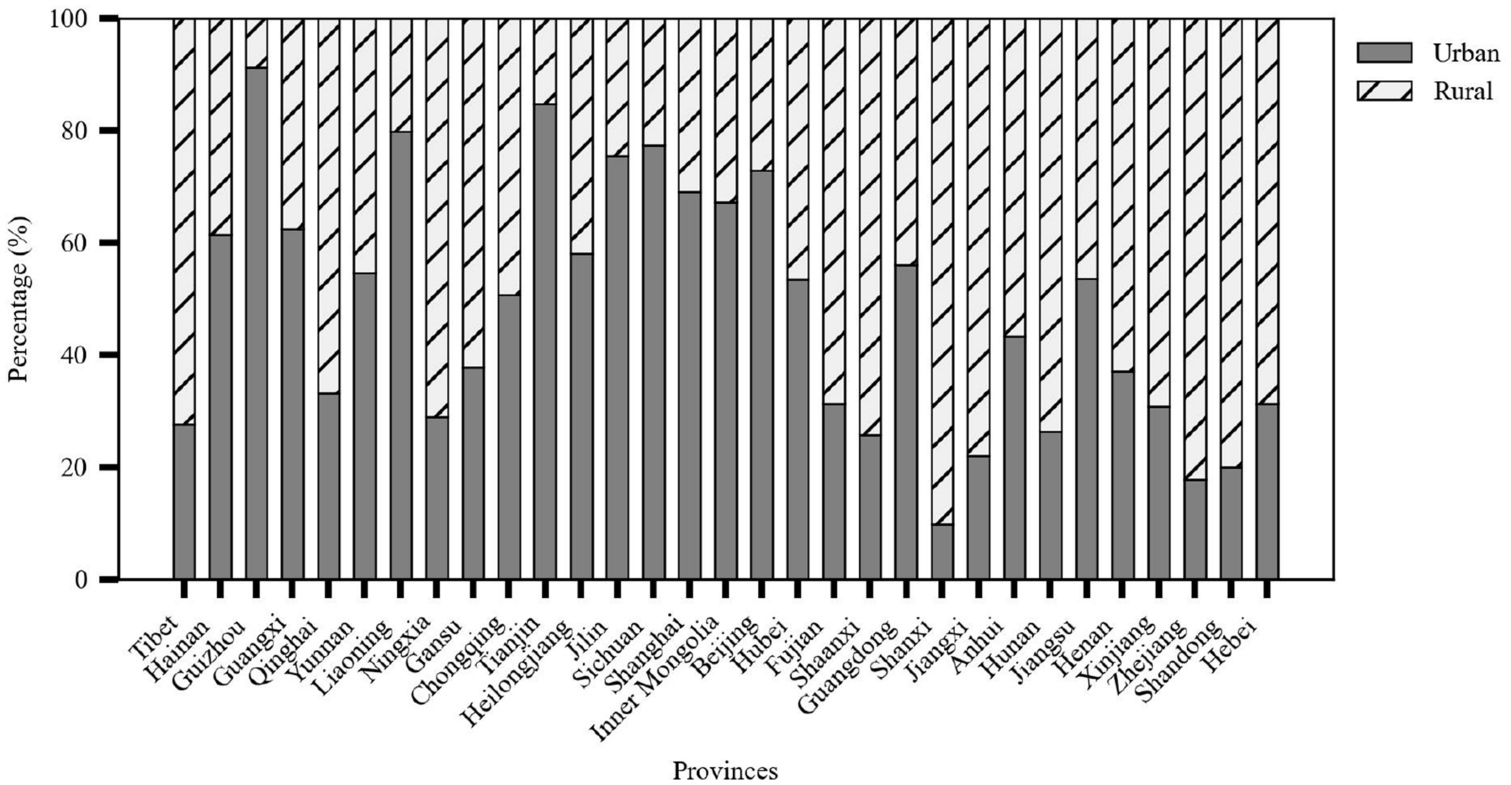
Stacked percentage chart of meal service facility types by province (source: original work).

Zhejiang Province had the highest number of rural older adult meal service facilities (9,609), followed closely by Shandong Province (9,638). This phenomenon may be explained by the vast geographical areas and large rural populations of these two provinces. Additionally, Guizhou Province had the highest proportion of urban older adult meal service facilities, reaching 91.26% as shown in Figure 5.

### 4.4 Meal service facility operational models

**Figure 6:**
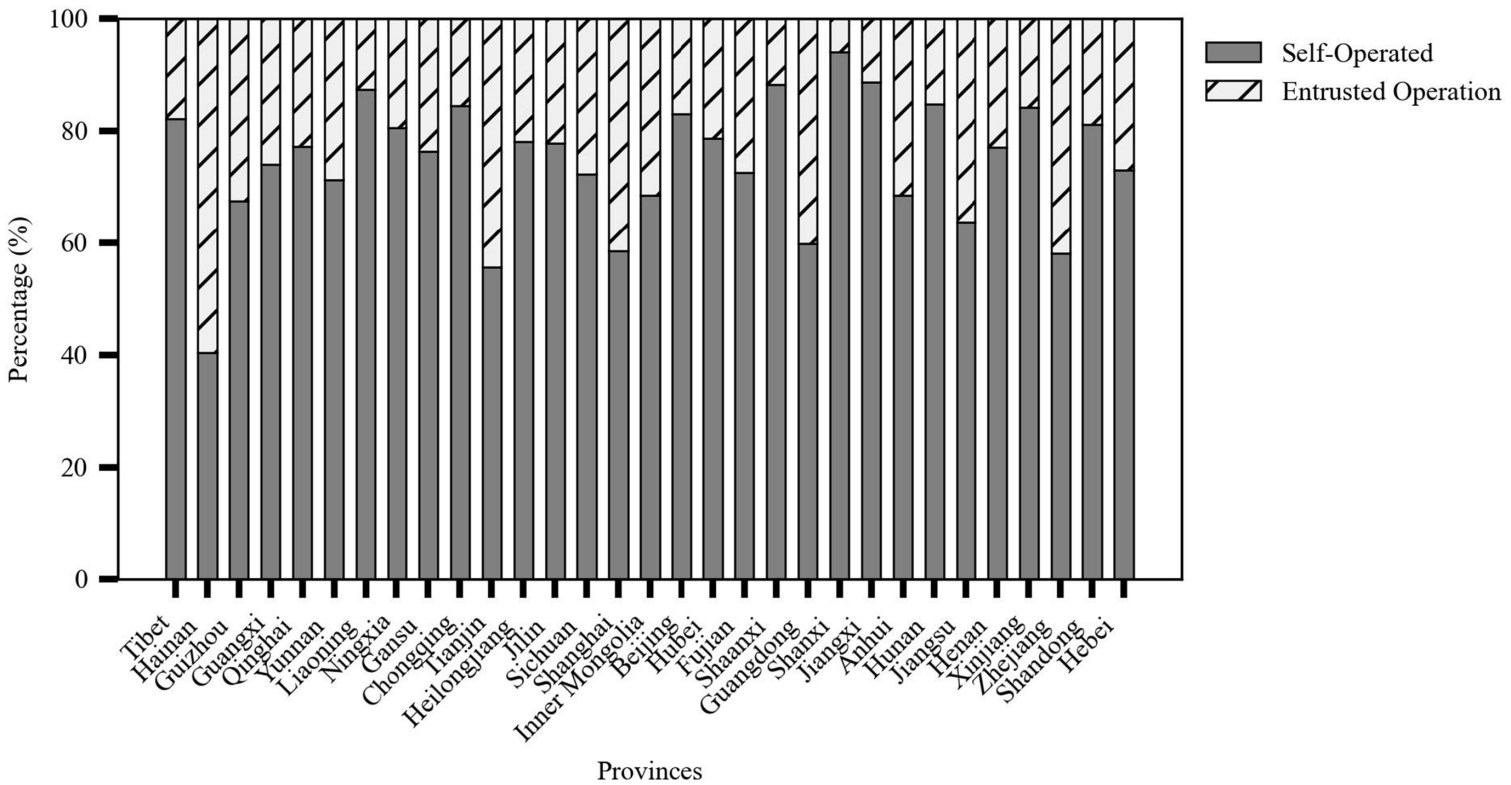
Stacked percentage bar chart of meal service facility operation models by province (source: original work).

In terms of operational models, Shanxi Province ranked first with a 94.03% self-operation rate, reflecting the dominant role of local governments in the construction of older adult care service facilities. In contrast, 41.52% of the older adult meal service facilities in Shanghai adopted the outsourced operation model, indicating the city’s preference for introducing third-party professional institutions to improve service efficiency and quality. Eastern coastal provinces such as Jiangsu, Zhejiang, and Shandong not only had a large number of older adult meal service facilities but also maintained a good balance between self-operation and outsourced operation as shown in Figure 6.

### 4.5 Meal service facility kitchen infrastructure

**Figure 7:**
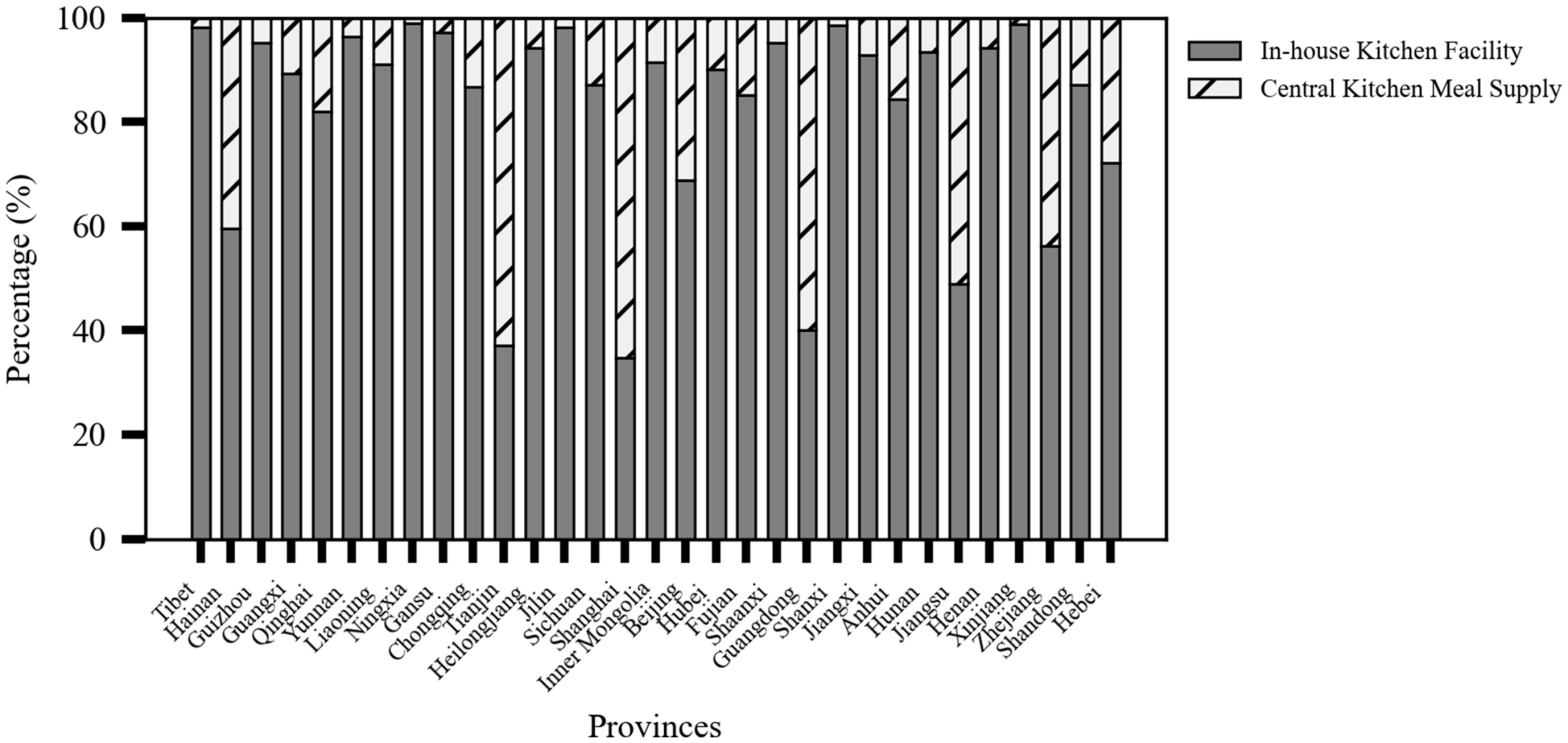
Stacked percentage bar chart of meal service facility kitchen infrastructure by province (source: original work).

Provinces with a high proportion of older adult meal service facilities equipped with on-site kitchens included Shanxi (98.57%), Jiangxi (92.81%), Shaanxi (95.13%), and Xinjiang (98.79%). These regions, due to their geographical environments, large rural populations, and policy support, tend to establish localized service facilities. In contrast, provinces with higher urbanization levels, such as Shanghai (65.21%), Tianjin (62.78%), Zhejiang (43.68%), and Guangdong (59.94%), rely more on central kitchen meal delivery models to improve efficiency and service standardization, catering to the needs of densely populated urban areas as shown in Figure 7.

## 5 Equilibrium analysis of older adult meal service facilities

### 5.1 Overall equilibrium

**Figure 8:**
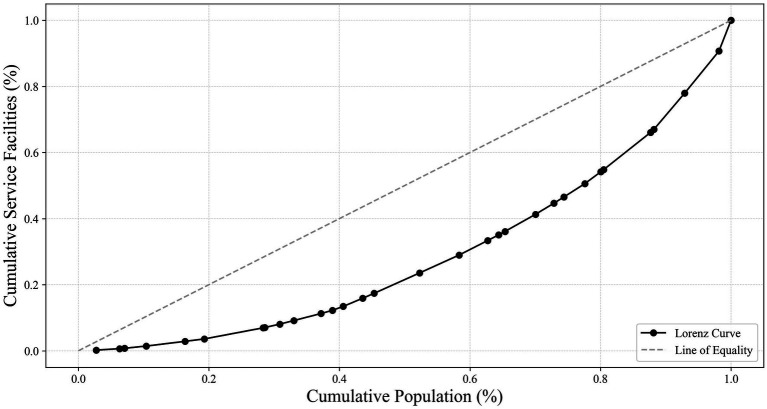
Lorenz curve of cumulative percentage of population aged 65 and above vs. cumulative percentage of older adult meal service facilities (source: original work).

This study used the Lorenz curve to visually depict the relationship between the cumulative percentage of the population aged 65 and above and the corresponding cumulative percentage of older adult meal service facilities, as shown in Figure 8. The results showed that the actual curve was below the line of absolute equality (45-degree line), especially in the early stages of the curve, revealing that older adults in some regions did not have sufficient access to meal service resources. As the cumulative population percentage increased, the curve gradually approached but never coincided with the line of absolute equality, reflecting an overall imbalance in resource allocation.

The Gini coefficient, a core indicator for measuring the degree of this imbalance, was calculated as G = 0.418 in this study, falling within the range of 0.4 to 0.5, which is generally considered to indicate moderate inequality. Specifically, this value suggested a lack of equity in the distribution of older adult meal service facilities nationwide. This imbalance warranted attention to better meet the needs of older adults in different regions.

Additionally, by calculating the Spatial Mismatch Index (SMI), the overall spatial mismatch level between older adult meal service facilities and the population aged 65 and above was found to be ∑SMI = 58.74. A higher ∑SMI value further demonstrates the irrationality of spatial allocation, with higher values indicating greater spatial mismatch between facilities and the target population, as well as a more unreasonable overall layout.

In summary, through the comprehensive analysis of the Lorenz curve, Gini coefficient, and Spatial Mismatch Index, this study revealed significant inequities in the distribution of older adult meal service facilities nationwide. These findings not only highlighted the issues of efficiency and fairness in current resource allocation but also provide critical data support and policy recommendations for optimizing older adult care services in the future.

### 5.2 Supply–demand equilibrium across provinces

To further reveal regional equity, this study explored the SMI values at the provincial level, as shown in Table 2, and calculated the SMI for each province.

**Table 2:** Spatial mismatch index by province.

Province	Total number of meal service facilities	Population aged 65 and over	SMI
Hebei	13,610	7,406,851	7.478
Xinjiang	9,944	2,602,854	7.456
Zhejiang	11,697	6,639,391	6.233
Shandong	12,046	10,141,960	4.076
Shanxi	3,855	3,472,502	1.144
Jiangxi	4,327	4,523,406	0.840
Shaanxi	3,576	3,959,372	0.537
Hunan	5,525	6,580,304	0.502
Ningxia	1,009	730,352	0.426
Beijing	2038	2,190,105	0.353
Qinghai	674	595,147	0.208
Tianjin	1,115	1,366,508	0.074
Anhui	4,675	6,132,445	0.024
Inner Mongolia	1840	2,400,476	0.019
Tibet	112	365,652	−0.155
Shanghai	1,604	2,491,627	−0.266
Jilin	1,262	2,343,387	−0.481
Fujian	2,634	4,190,866	−0.508
Hainan	114	1,044,910	−0.634
Jiangsu	5,789	8,541,960	−0.642
Gansu	1,049	2,469,648	−0.770
Henan	6,602	9,833,397	−0.797
Heilongjiang	1,173	3,067,745	−1.078
Chongqing	1,080	3,196,955	−1.257
Hubei	2,253	5,848,906	−2.040
Liaoning	778	4,189,819	−2.243
Guizhou	206	3,872,217	−2.553
Yunnan	711	4,681,732	−2.655
Guangxi	484	5,036,441	−3.118
Sichuan	1,529	8,383,635	−4.514
Guangdong	3,600	12,729,781	−5.659

Provinces such as Hebei (SMI = 7.478), Xinjiang (SMI = 7.456), and Zhejiang (SMI = 6.233) exhibited significantly positive SMI values, indicating that the number of older adult meal service facilities in these regions was relatively high compared to their population aged 65 and above, suggesting abundant resource allocation. This also implied that these regions have opportunities to further optimize resource allocation, ensuring that each facility is utilized efficiently while improving overall service efficiency.

Provinces like Guangdong (SMI = −5.659) and Sichuan (SMI = −4.514) showed significantly negative SMI values. Despite their large older adult populations, the number of older adult meal service facilities in these provinces is relatively insufficient, reflecting issues of uneven resource allocation. This situation could affect the quality of life and health of older adults, and it was recommended to take measures to increase facility supply and service coverage to better meet the needs of the local older adult population.

In economically developed regions such as Jiangsu (SMI = −0.642) and Shanghai (SMI = −0.266), despite having a certain advantage in the number of older adult meal service facilities, slight negative SMI values still appeared due to their large older adult populations. This phenomenon indicated that even in resource-rich areas, refined management and layout optimization should be emphasized to ensure that older adult care service facilities can efficiently and accurately meet the actual needs of the local older adult population.

In summary, by analyzing the SMI values of each province, this study was able to identify the characteristics and challenges of resource allocation for older adult meal service facilities in different provinces. This not only helped to understand whether the current service model can effectively respond to the needs of the older adult population but also provided a scientific basis for future policy-making and service optimization, aiming to achieve optimal resource utilization and service equity, thereby comprehensively improving the quality of life for older adults.

## 6 Factors influencing older adult meal service facilities

### 6.1 Selection of influencing factors

Among the influencing factors of older adult meal service facilities, Wang et al. and Chi suggested that the utilization rate of older adult care facilities is related to income inequality (35, 37). Hansen proposed that accessibility refers to the convenience of accessing urban opportunities, such as medical services (38). Song et al. found that geographical location, quality of care services, and proximity to hospitals are the main factors influencing older adults’ preferences for older adult care services (39). Sharma’s research on public transportation accessibility indicators provided a reference for this study (40).

This study was based on the concept of “geographical nature” and examined the spatial distribution characteristics of older adult meal service facilities from three dimensions: natural endowment (the first nature) proposed by Krugman (41), human activity choices (the second nature), and modern development factors (the third nature) proposed by Wang (42). The geographical area serves as the natural base that determines the physical boundary of facility coverage. The population scale (older adult population and dependency ratio) and economic level (GDP and per capita GDP) constitute the dynamic balance of demand and resources. The transportation network shapes the accessibility space. The degree of informatization (internet users) enhances operational efficiency through digital services, and policy response timeliness reflects institutional support. The interaction of these three natures revealed the deep mechanisms by which natural conditions, human choices, and modern technology jointly shape the spatial differentiation of facilities.

Therefore, this study selected 10 potential influencing factors that may affect the distribution of older adult meal service facilities, including: Population aged 65 and above, older adult dependency ratio, GDP, GDP per capita, Number of public buses and trolleys in operation, Number of medical institutions, Provincial area, Number of internet broadband subscribers, Number of people with higher education, Policy response time (in days), as shown in Table 3.

**Table 3:** Descriptive statistics of influencing factors.

Variables	*N*	mean	sd	min	max
Population aged 65 and above	31	454.9355	306.4022	36.6	1,273
Older adult dependency ratio	31	0.2174194	0.0521311	0.088	0.306
Higher education enrollment	31	8726.935	5691.836	462	25,369
GDP	31	40352.64	32815.65	2392.7	135673.2
GDP *Per Capita*	31	8.820368	3.800042	4.7867	20.0278
Number of public buses and electric vehicles in operation	31	22016.48	16158.88	874	63,952
Number of medical institutions	31	34.54145	24.50663	4.863	92.825
Broadband subscriber count	31	2052.606	1362.573	145.5	4,824
Provincial areas in China	31	31.00045	38.46969	0.634	166
Days for policy response	31	172.9355	86.63984	42	381

### 6.2 Factors affecting the quantity and coverage level of older adult meal service facilities

Multiple linear regression is capable of analyzing the impact of multiple factors (such as population, economy, and geography) on the distribution of older adult meal assistance facilities simultaneously. It provides a comprehensive analysis of the data rather than focusing on single-factor correlations. Based on the results of multiple linear regression analysis, this study delved into the key factors influencing the quantity and coverage of older adult meal service facilities, with detailed analysis results shown in Table 4.

**Table 4:** Results of multiple linear regression.

Independent variables and result	Total number of older adult meal service facilities			Coverage level of older adult meal service facilities		
Variables and intercept	*β*	*t*	*p*	*β*	*t*	*p*
Population aged 65 and above	−31.25**	−2.96	0.008	−0.00953*	−2.31	0.032
Older adult dependency ratio	−24560.6*	−2.41	0.026	−8.604*	−2.16	0.043
Higher education enrollment	0.266	−0.55	0.59	0.000247	−1.3	0.208
GDP	−0.218*	−2.58	0.018	−0.0000746*	−2.25	0.036
GDP *Per Capita*	425.5	−1.77	0.092	0.114	−1.21	0.242
Number of public buses and electric vehicles in operation	0.370***	−3.87	0.001	0.000068	−1.81	0.085
Number of medical institutions	98.04	−2.04	0.054	0.00853	−0.45	0.655
Broadband subscriber count	7.018**	−3.65	0.002	0.00204*	−2.71	0.013
Provincial areas in China	24.49	−1.92	0.069	0.0124*	−2.49	0.022
Days for policy response	8.837	−1.63	0.118	0.00229	−1.08	0.293
_cons	−2488.4	−0.72	0.482	0.322	−0.24	0.816

*p < 0.05, **p < 0.01, ***p < 0.001.

The population aged 65 and above, older adult dependency ratio, GDP, and the number of internet broadband subscribers all had significant impacts on the quantity and coverage level of older adult meal service facilities. Among these, the population aged 65 and above, older adult dependency ratio, and GDP showed a negative correlation with the quantity and coverage level of older adult meal service facilities. Specifically, the larger the population aged 65 and above, the fewer the number and lower the coverage level of older adult meal service facilities, indicating a mismatch between the current meal services and population demand, further highlighting the inequity in the distribution of older adult meal service facilities nationwide. A higher older adult dependency ratio was associated with fewer older adult meal service facilities, which may be related to the family structure in these provinces, where families are better able to care for older adults, thereby reducing the demand for meal services. Provinces with higher GDP tended to have fewer older adult meal service facilities, possibly because older adults in these regions prefer market-based older adult care services over government-provided meal services, which may also lead to lower coverage level of older adult meal service facilities.

On the other hand, the number of internet broadband subscribers showed a positive correlation with the quantity and coverage level of older adult meal service facilities, suggesting that the widespread use of the internet may provide older adults with more convenient meal ordering and delivery services, thereby promoting the development of older adult meal service facilities.

Additionally, the number of public buses and trolleys in operation had a significant positive impact on the quantity of older adult meal service facilities, likely because convenient transportation makes it easier for older adults to access meal services. Furthermore, provincial area has a significant positive impact on the coverage level of older adult meal service facilities, as larger provinces with more dispersed populations may achieve higher coverage level even with fewer facilities. This study found that the number of people with higher education, the number of medical institutions, and policy response time have no significant impact on the quantity and coverage level of older adult meal service facilities.

## 7 Discussion and conclusion

### 7.1 Discussion

By constructing the Lorenz curve and calculating the Gini Coefficient and Spatial Mismatch Index, this study identified significant inequities in the allocation of resources between the population aged 65 and above and older adult meal service facilities nationwide. This finding aligns with existing research on the inequity of older adult care services in China (7, 8). At the provincial level, the distribution of supply and demand for older adult meal service facilities is highly uneven. For instance, Hebei exhibited a severe oversupply of facilities, likely attributable to its proximity to Beijing, where high land prices and supportive policies for older adult care services extend into Hebei. In contrast, Guangdong and Sichuan face significant undersupply, possibly due to intra-provincial infrastructure disparities (43, 44).

In studies on the equity of older adult care services, regions with larger older adult populations, higher older adult dependency ratios, or better economic conditions tend to have more developed older adult care services (45). However, this study found a negative correlation between the availability of older adult meal service facilities and regions exhibiting these characteristics, further highlighting the inequitable development of older adult meal service facilities. Possible reasons for this phenomenon include: (1) In regions with dense older adult populations, despite high demand for older adult care services, resource allocation pressures may result in a relative undersupply of meal services; (2) In economically developed regions, despite having stronger older adult care infrastructures, older adults and their families often opt for market-based catering services instead of public older adult meal service facilities. This trend may indicate that local governments and communities have not effectively promoted the benefits of public older adult meal services to older adults and their families. As a result, the actual demand for such services among older adults remains under-realized and inadequately addressed.

This study confirmed a strong link between public transportation scale and older adult meal service development, supporting Cheng et al.’s findings on transport accessibility shaping care facility distribution (46). Robust transit systems enhanced older adult mobility and staff efficiency, yet Zhang et al. highlighted persistent barriers: physical limitations, digital gaps, and economic constraints (47). Concurrently, rising internet usage correlated with expanded service coverage, driving innovations like online meal delivery and telemedicine integration. As Xu et al. proposed, such digital empowerment improved healthcare accessibility for seniors while addressing multidimensional exclusion, necessitating integrated infrastructure and age-friendly technological adaptation (48).

Although this study revealed the inequitable distribution of older adult meal service and analyzed its influencing factors, some limitations remain that require attention in future research. The provincial-level analysis, provides a primarily macro-level overview and does not deeply explore the specific causes of disparities across provinces. Future research could refine the scope of data collection to more comprehensively reflect the actual distribution and demand for older adult meal service facilities. Additionally, future studies could incorporate time-series data to dynamically analyze trends in the supply–demand relationship of these facilities, providing more timely and targeted scientific evidence for policy-making. Through multidimensional and multi-method comprehensive research, the intrinsic patterns of older adult meal service development can be more fully revealed, offering stronger support for promoting regional equity and optimizing resource allocation.

## 8 Conclusion

This study utilized data from the National Elderly Care Information Platform to conduct a comprehensive statistical analysis of older adult meal service facilities. The results revealed significant differences in the development direction of older adult meal services among provinces. Through analyzing the supply and demand distribution of older adult meal service facilities across provinces, this study found a considerable mismatch between the current supply of these facilities and the actual needs of the older adult population. Specifically, Guangdong province faces the most acute shortage in terms of the relationship between older adult meal service facilities and its older adult population, indicating a clear inadequacy in resource allocation. This phenomenon is prevalent nationwide, with significant variations among provinces, further exacerbating the imbalance in older adult meal services. In terms of influencing factors, there was a notable negative correlation between the number and coverage level of older adult meal service facilities and the older adult population size, which further confirmed the uneven distribution of older adult meal service facilities nationwide. By dissecting the characteristics of supply–demand distribution of older adult meal services and their influencing factors, this study provided scientific evidence for optimizing the balanced allocation of national older adult meal service resources and formulating targeted support policies. It held substantial practical significance for narrowing regional service disparities and enhancing the well-being of the older adult population.

## Data Availability

Publicly available datasets were analyzed in this study. This data can be found here: https://yanglao.mca.gov.cn and https://data.stats.gov.cn.

## Footnotes

^1^ https://yanglao.mca.gov.cn

^2^ https://data.stats.gov.cn

## References

[1] Ministry of Civil Affairs of the People's Republic of China, National Working Commission on Aging. 2023 national report on the development of aging affairs. (2023). Available online at:https://www.gov.cn [Accessed December 25, 2024].

[2] Bai R Liu Y Zhang L Dong W Bai Z Zhou M. Projections of future life expectancy in China up to 2035: a modelling study. Lancet Public Health. (2023) 8:e915–22. doi: 10.1016/S2468-2667(22)00338-3, PMID: 37004714 PMC10188127

[3] Bai C Lei X. New trends in population aging and challenges for China’s sustainable development. China Econ J. (2019) 13:3–23. doi: 10.1080/17538963.2019.1700608

[4] Feng Z Glinskaya E Chen H Gong S Qiu Y Xu J. Long-term care system for older adults in China: policy landscape, challenges, and future prospects. Lancet (London, England). (2020) 396:1362–72. doi: 10.1016/S0140-6736(20)32136-X, PMID: 34338215

[5] Chen S Si Y Hanewald K Li B Bateman H Dai X. Disease burden of ageing, sex and regional disparities and health resources allocation: a longitudinal analysis of 31 provinces in mainland China. BMJ Open. (2022) 12:e064641. doi: 10.1136/bmjopen-2022-064641, PMID: 36385040 PMC9670959

[6] Bao J Zhou L Liu G Tang J Lu X Cheng C. Current state of care for the elderly in China in the context of an aging population. Biosci Trends. (2022) 16:107–18. doi: 10.5582/bst.2022.01068, PMID: 35431289

[7] Zhang Z Mao Y Shui Y Deng R Hu Y. Do Community home-based elderly care services improve life satisfaction of Chinese older adults? An empirical analysis based on the 2018 CLHLS dataset. Int J Environ Res Public Health. (2022) 19:15462. doi: 10.3390/ijerph192315462, PMID: 36497536 PMC9738417

[8] He Y Wei B Li Y. The impact of using community home-based elderly care services on older adults' self-reported health: fresh evidence from China. Front Public Health. (2023) 11:1257463. doi: 10.3389/fpubh.2023.1257463, PMID: 37799160 PMC10549933

[9] Ministry of Civil Affairs of the People's Republic of China. (2023). China releases national standard for elderly home care. Xinhua News Agency. Available online at:https://english.www.gov.cn/news/202310/20/content_WS653281c7c6d0868f4e8e07e1.html [Accessed December 25, 2024]

[10] Maslow AH. A theory of human motivation. Psychol Rev. (1943) 50:370–96. doi: 10.1037/h0054346

[11] Wang X Qi J Zhang K Xie H Wu X. The joy of eating: how eating experiences enhance the well-being of older adults. Front Public Health. (2024) 12:1438964. doi: 10.3389/fpubh.2024.1438964, PMID: 39314795 PMC11417023

[12] Hu FB. Diet strategies for promoting healthy aging and longevity: an epidemiological perspective. J Intern Med. (2024) 295:508–31. doi: 10.1111/joim.13728, PMID: 37867396 PMC10939982

[13] Walton K do Rosario VA Pettingill H Cassimatis E Charlton K. The impact of home-delivered meal services on the nutritional intake of community living older adults: a systematic literature review. J Hum Nutr Diet. (2020) 33:38–47. doi: 10.1111/jhn.12690, PMID: 31266095

[14] Wang X Liu M Li Y Guo C Yeh CH. Community canteen services for the rural elderly: determining impacts on general mental health, nutritional status, satisfaction with life, and social capital. BMC Public Health. (2020) 20:230. doi: 10.1186/s12889-020-8305-9, PMID: 32059652 PMC7023764

[15] SeoSKimOYAhnJ. Healthy eating exploratory program for the elderly: low salt intake in congregate meal service. J Nutr Health Aging. (2016) 20:316–24. doi: 10.1007/s12603-015-0622-9, PMID: 26892581

[16] Thomas KS Parikh RB Zullo AR Dosa D. Home-delivered meals and risk of self-reported falls: results from a randomized trial. J Appl Gerontol. (2018) 37:41–57. doi: 10.1177/0733464816675421, PMID: 27798291 PMC6620777

[17] Thomas KS Akobundu U Dosa D. More than a meal? A randomized control trial comparing the effects of home-delivered meals programs on participants' feelings of loneliness. J Gerontol B Psychol Sci Soc Sci. (2016) 71:1049–58. doi: 10.1093/geronb/gbv111, PMID: 26613620

[18] Middleton G Patterson KA Muir-Cochrane E Velardo S McCorry F Coveney J. The health and well-being impacts of community shared meal programs for older populations: a scoping review. Innov Aging. (2022) 6:igac–068. doi: 10.1093/geroni/igac068 PMC9795837 36588625

[19] Ministry of Civil Affairs of the People's Republic of China. (2023). Meal services for elderly to expand nationwide. China Daily. Available online athttps://english.www.gov.cn/news/202310/20/content_WS653281c7c6d0868f4e8e07e1.html

[20] Dickinson A Wills W. Meals on wheels services and the food security of older people. Health Soc Care Community. (2022) 30:e6699. doi: 10.1111/hsc.14092, PMID: 36300541 PMC10092458

[21] Gualtieri MC Donley AM Wright JD Vega SS. Home delivered meals to older adults: a critical review of the literature. Home Healthc Now. (2018) 36:159–68. doi: 10.1097/NHH.0000000000000665, PMID: 29722706

[22] Hoerr KA Francis SL Margrett JA Peterson M Franke WD. Promoting the congregate meal program to the next generation of rural-residing older adults. J Nutr Gerontol Geriatr. (2016) 35:113–23. doi: 10.1080/21551197.2016.1163313, PMID: 27153251

[23] Nkurunziza M Mchiza ZJ-R Zembe Y. Meals on wheels: promoting food and nutrition security among older persons in Cape Town, South Africa. Int J Environ Res Public Health. (2023) 20:2561. doi: 10.3390/ijerph20032561, PMID: 36767923 PMC9915356

[24] Zhang L Wei L Zhang W Fang Y. Bridging the gap: coordinating equity and efficiency in older people care resource allocation in China. BMC Geriatr. (2024) 24:165. doi: 10.1186/s12877-024-04696-w, PMID: 38365604 PMC10874015

[25] Yu Q Zhang T Jiang L Jia Y Dong Y Luo L. Equity analysis of older adult resource allocation in China. Front Public Health. (2024) 12:1411054. doi: 10.3389/fpubh.2024.1411054, PMID: 39071147 PMC11273785

[26] Smith A. An inquiry into the nature and causes of the wealth of nations. Read Econ Sociol. (2002) 6-17. doi: 10.1002/9780470755679.ch1

[27] Marshall A. Principles of economics. London: Macmillan and Co (1890).

[28] Gastwirth JL. A general definition of the Lorenz curve. Econometrica. (1971) 39:1037–9. doi: 10.2307/1909675

[29] Kakwani NC. Applications of Lorenz curves in economic analysis. Econometrica. (1977) 45:719–27. doi: 10.2307/1911684

[30] Gastwirth JL. The estimation of the Lorenz curve and Gini index. Rev Econ Stat. (1972) 54:306–16. doi: 10.2307/1937992

[31] Du J Yang Z Wang H Yang G Li S. Spatial–temporal matching characteristics between agricultural water and land resources in Ningxia, Northwest China. Water. (2019) 11:1460. doi: 10.3390/w11071460

[32] Fan Y Allen R Sun T. Spatial mismatch in Beijing, China: implications of job accessibility for Chinese low-wage workers. Habitat Int. (2014) 44:202–10. doi: 10.1016/j.habitatint.2014.06.002

[33] Zhou Y Zhao K Han J Zhao S Cao J. Geographical pattern evolution of health resources in China: Spatio-temporal dynamics and spatial mismatch. Tropical Med Infect Dis. (2022) 7:292. doi: 10.3390/tropicalmed7100292, PMID: 36288033 PMC9609797

[34] Uyanık GK Güler N. A study on multiple linear regression analysis. Procedia Soc Behav Sci. (2013) 106:234–40. doi: 10.1016/j.sbspro.2013.12.027

[35] Wang Y Feng X Chai Y Chen K Yang S Li W. Coupling coordination relationship between health resource allocation and regional economic development: an empirical study based on five provinces in eastern China. Front Public Health. (2024) 12:1513188. doi: 10.3389/fpubh.2024.1513188, PMID: 39737454 PMC11683099

[36] Penghui X Xicang Z Haili L. Direct and indirect effects of health expenditure on economic growth in China. Eastern Mediterranean health journal = La revue de sante de la Mediterranee orientale = al-Majallah al-sihhiyah li-sharq al-mutawassit. (2022) 28:204–12. doi: 10.26719/emhj.22.007, PMID: 35394052

[37] Chi Z Lun H Ma J Zhou Y. Income inequality and healthcare utilization of the older adults-based on a study in three provinces and six cities in China. Front Public Health. (2024) 12:1435162. doi: 10.3389/fpubh.2024.1435162, PMID: 39114522 PMC11303323

[38] Hansen WG. How accessibility shapes land use. J Am Inst Plann. (1959) 25:73–6. doi: 10.1080/01944365908978307

[39] Song S Wang D Zhu W Wang C. Study on the spatial configuration of nursing homes for the elderly people in Shanghai: based on their choice preference. Technol Forecast Soc Change. (2020) 152:119859. doi: 10.1016/j.techfore.2019.119859

[40] Sharma G Patil GR. Public transit accessibility approach to understand the equity for public healthcare services: a case study of greater Mumbai. J Transp Geogr. (2021) 94:103123. doi: 10.1016/j.jtrangeo.2021.103123

[41] Krugman P. First nature, second nature, and metropolitan location. J Reg Sci. (1993) 33:129–44. doi: 10.1111/j.1467-9787.1993.tb00217.x

[42] Wang Z. Geographical nature: the breakthrough and challenges of the Hu Huanyong line. Explor Contention. (2016):43–7.

[43] Liao FH Wei YD. Dynamics, space, and regional inequality in provincial China: a case study of Guangdong province. Appl Geogr. (2012) 35:71–83. doi: 10.1016/j.apgeog.2012.05.003

[44] Wang X Yang H Duan Z Pan J. Spatial accessibility of primary health care in China: a case study in Sichuan Province. Soc Sci Med. (2018) 209:14–24. doi: 10.1016/j.socscimed.2018.05.023, PMID: 29778934

[45] Wu H-C Tseng M-H. Evaluating disparities in elderly community care resources: using a geographic accessibility and inequality index. Int J Environ Res Public Health. (2018) 15:1353. doi: 10.3390/ijerph15071353, PMID: 29954156 PMC6068710

[46] Cheng L Yang M De Vos J Witlox F. Examining geographical accessibility to multi-tier hospital care services for the elderly: a focus on spatial equity. J Transp Health. (2020) 19:100926. doi: 10.1016/j.jth.2020.100926

[47] Zhang N Yang Q. Public transport inclusion and active ageing: a systematic review on elderly mobility. J Traffic Transp Eng (Engl Ed). (2024) 11:312–47. doi: 10.1016/j.jtte.2024.04.001

[48] Xu H e B Chang CH. Empowering the care of older adults through the use of technology. Work Aging Retire. (2024) 10:1–5. doi: 10.1093/workar/waad030 38196828 PMC10772963

